# Myocarditis Associated with Influenza A H1N1pdm2009

**DOI:** 10.1155/2012/351979

**Published:** 2012-12-17

**Authors:** Akira Ukimura, Hidetoshi Satomi, Yukimasa Ooi, Yumiko Kanzaki

**Affiliations:** ^1^Department of General Internal Medicine, Osaka Medical College, Takatsuki 569-8686, Japan; ^2^Third Department of Internal Medicine, Osaka Medical College, Takatsuki 569-8686, Japan

## Abstract

Acute myocarditis is a well-known complication of influenza infection. The frequency of myocardial involvement in influenza infection varies widely, with the clinical severity ranging from asymptomatic to fulminant varieties. The worst cases can result in death due to impaired cardiac function, although such fulminant myocarditis associated with influenza infection is rare, as shown by previous papers. Following the 2009 influenza pandemic, we reported on the clinical features of a cohort of 15 patients in Japan with H1N1pdm2009 myocarditis. In our subsequent survey of the literature for case reports or series of patients with myocarditis associated with H1N1pdm2009, we identified 58 detailed cases. We discuss here the high prevalence of fulminant myocarditis (36/58, 62%) among patients reported to have myocarditis associated with H1N1pdm2009. Mechanical circulatory support was required in 17 of the patients with fulminant myocarditis, 13 of whom recovered. We stress the need for increased awareness of influenza-associated myocarditis; such knowledge will facilitate earlier diagnosis and treatment of this fatal complication during future influenza pandemics.

## 1. Introduction

Acute myocarditis is a well-known complication of influenza infection. The clinical expression varies from asymptomatic to fulminant myocarditis, which can result in severe hemodynamic dysfunction, necessitating high-dose catecholamines and mechanical circulatory support [1–11]. Pathogens frequently associated with myocarditis include coxsackievirus and adenovirus; fulminant myocarditis resulting from influenza A viral infection is rare, as shown by previous literature [1–23]. Our interest in influenza-associated myocarditis follows from our experience with the influenza pandemic of 2009 [3, 24–30]. We surveyed the literature for case reports and series involving myocarditis associated with H1N1pdm2009, and identified 58 patients with such a diagnosis [3, 31–62]. In the present study, we review the clinical, laboratory, and pathologic characteristics of these 58 patients and theorize about the pathogenesis of influenza myocarditis [63–68].

## 2. Cardiac Involvement of Influenza Infection  before the 2009 Pandemic

Myocarditis was a common and sometimes fatal complication of influenza infection in the pandemics of the previous century [1–7]. Small autopsy-based studies on fatal cases revealed a complication rate of focal to diffuse myocarditis of 39.4% during the 1957 Asian influenza pandemic and 48% during the Spanish influenza pandemic [4–6]. All of these fatal cases with myocarditis also had severe pneumonia and multiple organ involvement. Thus, myocarditis is likely to be a terminal complication of pandemic influenza infection.

On the other hand, while many people are affected by seasonal influenza every year, complications in nonrespiratory tissues (e.g., encephalopathy, myocarditis, and myopathy) occur only occasionally [1–7]. The frequency of myocardial involvement in influenza infection varies (0–10%) depending on the diagnostic criteria, and fulminant myocarditis associated with seasonal influenza infection is rare, as shown in previous papers [1–4, 9, 12, 13, 15–23]. Indeed, only two (2/505, 0.4%) myocarditis cases were reported in 505 children admitted with laboratory-confirmed influenza during the 2003/2004 season in Canada [16].

Only rarely are influenza viral antigens or genetic material detected in the myocardium. There has been only one case report in which seasonal influenza A RNA was detected in a myocardial biopsy [15]. Miura et al. detected viral antigen in the myocardium using immunohistochemical staining on an autopsied heart [18]. Bowles et al. screened endomyocardial biopsy samples from 624 patients with clinically defined myocarditis using PCR for various viral genes. Among 239 samples that tested positive for viral genes, adenovirus was detected in 142 samples, enterovirus in 85 samples, and influenza A in only five samples (0.8%) [12]. Caforio et al. screened endomyocardial biopsy samples from 120 patients with histologically proven myocarditis using PCR to detect various viral genes. Among 31 samples that tested positive for viral genes, none contained influenza A or B virus (0%) [13]. Thus, the myocardial toxigenicity of the seasonal influenza virus seems to be rather weak.

## 3. Myocarditis Associated with Influenza  H1N1pdm2009 in Japan

The Ministry of Health, Labor and Welfare (MHLW) of Japan confirmed only 198 deaths among about 20.61 million patients infected with influenza A H1N1pdm2009 in the pandemic season in Japan. They also confirmed that 15 of these deaths resulted from myocarditis associated with this pandemic strain [28, 29]. We previously reported 15 H1N1pdm2009 myocarditis patients and demonstrated their clinical features by conducting a cross-sectional national survey with assistance from all members of the Japanese Circulation Society (JCS) in the 2009/2010 influenza season [31]. Myocarditis was diagnosed using the Guidelines for Diagnosis and Treatment of Myocarditis (JCS 2009) [8]. Seven (47%) of the 15 myocarditis patients had no baseline disease. Myocarditis was proved by endomyocardial biopsy in six patients. Histological findings in these six patients included myocarditis with degenerated myocytes, infiltration of lymphocytes (ranging from mild to moderate, but not severe), and interstitial edema. We demonstrated a high prevalence of fulminant myocarditis with fatal arrhythmias and/or varying degrees of cardiogenic shock among the majority (10/15, 67%) of patients with myocarditis. Mechanical circulatory support with intra-aortic balloon pumping (IABP) and/or percutaneous cardiopulmonary support (PCPS) was emergently required in 10 patients. Eight of these 10 patients were successfully rescued with mechanical circulatory support, while the remaining two patients died. We demonstrated that, along with pneumonia and encephalopathy, myocarditis was an important cause of clinical deterioration in patients infected with the pandemic H1N1pdm2009 virus in Japan.

## 4. Myocarditis Associated with Influenza  H1N1pdm2009 in the World

We reviewed the data of 58 patients (28 males and 30 females; mean age 32 years) with myocarditis associated with H1N1pdm2009 worldwide [3, 31–62] and identified a high prevalence of fulminant myocarditis (36/58, 62%) among them. The characteristics of these 58 myocarditis patients are summarized in Table 1. The mean age (32 years) of myocarditis patients associated with H1N1pdm2009 influenza was lower than the age of patients with seasonal influenza in the present study, indicating an age shift to a younger population in myocarditis patients during the pandemic [27–30, 32]. We speculate that the pathological mechanism of influenza myocarditis differs depending on the pathogen, and may depend on host immunity, as indicated by anti-H1N1pdm2009 titers.

Forty-two percent of these myocarditis patients had no baseline disease, and 23% had preexisting lung disease. The number of female patients was larger than the number of male patients, although general acute myocarditis is more common in males [69, 70]. Further, although pregnancy is reported to be a risk factor for deterioration of pandemic influenza infection, only one of the women in this paper was pregnant [38]. The mean interval from influenza onset to cardiac involvement was 5.4 days. Cardiac symptoms developed on the first to third day of sickness in 51% of myocarditis patients. Thirteen (24%) of the 58 cases were complicated by pneumonia. Most of these patients exhibited electrocardiogram (ECG) abnormalities, such as ST elevation (34%) and inverted T waves (24%). Fatal arrhythmias, such as ventricular fibrillation, ventricular tachycardia, and complete AV block, were recorded on the first day of hospitalization in 22% of the cases. Echocardiography revealed diffuse or focal left ventricular wall motion abnormalities in 90% of the patients. Mean ejection fraction was 25 ± 11%. Mortality rate was 24% (14 deaths/58 patients). Coronary studies were performed in 41% of these patients (64% of adult patients), all of which were normal with the exception of one case with a chronic total lesion. Myocarditis was proved by endomyocardial biopsy and/or autopsy in 14 patients. Myocardial biopsy did not contribute to the diagnosis of myocarditis in several cases. In the six patients in whom endomyocardial biopsy was performed, the pathological findings were mild even in clinically defined fulminant myocarditis patients, compared with general myocarditis patients reported in previous papers [71, 72]. Although immunohistology has been acknowledged to have a substantially higher sensitivity, we did not have detailed information on the immunohistological analysis of biopsies [73, 74].

Cardiovascular magnetic resonance imaging (CMR) was used as the diagnostic tool in several cases with pericardial/myocardial involvement during H1N1pdm2009 infection [47–50, 55, 58, 73–75]. A neuraminidase inhibitor (either oseltamivir, zanamivir, or peramivir) was used in 85% of the cases. A left ventricular assist device (LVAD) or PCPS was used in 10 cases, and IABP was used in 11 cases. Extracorporeal lung assist with extracorporeal membrane oxygenation (ECMO) was used in 12 cases. Mechanical circulatory support (PCPS or LVAD and/or IABP) was used in 17 of the patients with fulminant myocarditis, 13 of whom were rescued. Patchy hemorrhage was demonstrated in three autopsy cases. Reverse transcriptase polymerase chain reaction (RT-PCR) for H1N1pdm2009 from heart specimens tested positive in four cases [44, 51, 53, 58].

In the 2009 pandemic, the rate of cardiac complications seemed to be higher than that reported for seasonal influenza A virus infection. Randolph et al. reported that acute myocarditis associated with H1N1pdm2009 (1.4% of 838 cases) was an independent risk factor for death in children (<21 years old) admitted to a PICU in the USA [61]. Bratincsák et al. reported four patients with myocarditis associated with H1N1pdm2009 within a 30-day period in 2010 and suggested that H1N1pdm2009 virus might be more commonly associated with myocarditis than seasonal influenza virus [58]. Zheng et al. reported finding seven children (5%) with complicated myocarditis among 148 children hospitalized with influenza H1N1pdm2009 infection in China [62]. Shin et al. analyzed a group of 30 critically ill pediatric patients in Korea and reported that the most common causes of death were encephalopathy (four children) and myocarditis (four children) [63]. Martin et al. examined a cohort of 123 hospitalized patients infected with H1N1pdm2009 and reported that six patients (4.9%) had either new or worsened left ventricular dysfunction. They concluded that reversible cardiac dysfunction is a relatively common complication associated with H1N1pdm2009 [60]. Thus, the frequency of cardiac involvement in influenza virus infection is likely elevated with influenza H1N1pdm2009 compared to seasonal influenza.

## 5. Theories of Pathogenesis of Influenza  Myocarditis

It is well known that coxsackieviruses present a high affinity for cardiac myocytes [9, 12–14]. There is a distinct difference in the pathological findings between myocarditis associated with influenza A virus and myocarditis associated with coxsackieviruses [1–14]. The pathological effects of influenza viral myocarditis in humans and mice are reportedly milder and are more localized than those seen in coxsackievirus myocarditis [8, 9, 14]. Kotaka et al. reported that murine influenza myocarditis was histologically mild and brief in duration compared to coxsackievirus B3 myocarditis [64]. Electron microscopic findings of the heart from a murine influenza myocarditis model showed many infiltrating lymphocytes directly attached to the cardiac myocytes. Nonetheless, the affinity of the influenza virus for cardiac myocytes appears to be low.

Pan et al. investigated the molecular mechanism of myocarditis associated with the influenza virus and revealed the importance of trypsin induction and increased production of matrix metalloproteinase (MMP) and proinflammatory cytokines in the pathogenesis of acute myocarditis [65–68]. Pan et al. also showed that inhibition of trypsin suppressed viral replication, upregulated of MMPs and cytokines, and significantly improved the cardiac function of mice infected with influenza A virus [65–68]. Teijaro et al. revealed immune cell infiltration and cytokine production as distinct events, both of which are orchestrated by endothelial cells [68]. Beside the direct effect of influenza virus infection, proinflammatory cytokines and endothelial cell dysfunction are thought to contribute to the pathogenesis of severe clinical features, including severe cardiac dysfunction and encephalopathy in patients infected with influenza virus [65–68].

Calore et al. observed perivascular hemorrhage of the brain in five of six autopsies of H1N1pdm2009 cases; focal myocarditis was also observed in one case [45]. They suggested that hemorrhagic lesions in the brain might be due to vascular lesions or to an increase in endothelial permeability. Edler et al. demonstrated that an autopsy of a fulminant myocarditis case showed small patch-shaped hemorrhages on the top of the heart and a florid myocarditis with marked mixed-cell infiltrates; H1N1pdm2009 virus was detected in the brain and heart by RT-PCR [53]. RT-PCR from the myocardium showed positive results in four of the patients surveyed in the present paper. Thus, although the pathogenesis of influenza-associated myocarditis remains unclear, the literature suggests that endothelial dysfunction may be important in the pathogenesis of myocarditis and encephalopathy associated with influenza virus.

## 6. Diagnosis of Myocarditis Associated with  Influenza A Virus

In the present paper, chest pain or worsening dyspnea was a common symptom in patients with myocarditis associated with H1N1pdm2009. Cardiac symptoms (e.g., dyspnea, cough, palpitation, and impaired consciousness) developed from the first sick day to the third sick day in 51% of patients. On the other hand, cardiac dysfunction reportedly developed after recovery from flu-like symptoms in two patients. Since cardiac dysfunction progressed rapidly in H1N1pdm2009 myocarditis, early diagnosis and prompt treatment of acute myocarditis with heart failure are required in patients with influenza infection during the pandemic season.

The ECG is a sensitive and convenient tool for diagnosis of myocarditis. ST elevation, T inversion, and conduction block are frequently observed. The ECG and echocardiogram must be repeated for the diagnosis of myocarditis in patients with suspected myocarditis; ECG monitoring is also useful to detect fatal arrhythmias [7, 8]. Myocarditis can be confirmed by observation of transient wall thickening, reduced wall motion, and reduced cardiac chamber size in addition to pericardial effusion on echocardiography [8, 76]. Erden et al. reported that tissue Doppler echocardiography is useful to detect subclinical cardiac dysfunction [77]. It is also important to perform echocardiography to distinguish fulminant myocarditis, which is a lethal disease, from acute myocarditis. Felker et al. reported that patients with fulminant myocarditis had near normal diastolic dimensions with increased septal thickness, while those with acute myocarditis had increased diastolic dimensions with normal septal thickness [11].

Myocarditis is confirmed by the findings of transient elevation of creatinine kinase (CPK), the MB form of creatinine kinase (CPK-MB), and cardiac troponin. Brown et al. reported the usefulness of troponin as a diagnostic test in patients in the pediatric emergency department who report chest pain, although troponin is not useful for excluding cardiac ischemic disease in adults [78]. Erden et al. and Sahin et al. reported acute myocarditis mimicking acute myocardial infarction associated with H1N1pdm2009 infection, with chest pain and ST elevation, suggesting that coronary artery disease should be excluded in cases with severe chest pain [35, 42]. Coronary artery disease was excluded by coronary study in 64% of the adults in this paper. For even more definitive diagnosis, endomyocardial biopsy should be performed after the coronary lesion has been excluded, although it is not essential for the clinical diagnosis of myocarditis. However, even if the results of cardiac biopsy are negative, the presence of myocarditis cannot be excluded due to the possibility of sampling error. Baccouche et al. reported that CMR and endomyocardial biopsy have good diagnostic performance as single techniques in patients with Troponin-I-positive acute chest pain in the absence of coronary artery disease [74]. Liu and Yan observed that CMR, a new technique, is helpful for the detection of myocarditis, because CMR can visualize the entire myocardium [75]. Gutbertet et al. reported that although CMR imaging may be helpful in noninvasively detecting intramyocardial inflammation, it fails to detect viral persistence [73]. Takeuchi et al. reported that MRI might be more useful than invasive cardiac biopsy for diagnosing H1N1pdm2009 myocarditis and for estimating the activity and severity of inflammation [49]. Mavrogeni and Manoussakis recommended CMR for its sensitivity in detecting pericardial/myocardial involvement during H1N1pdm2009 infection, especially if echocardiographic evaluation is negative [55]. Thus, CMR may be useful for the diagnosis of influenza virus myocarditis, because the affinity of influenza virus for cardiac myocytes appears to be low, identifying the intramyocardial inflammation of influenza myocarditis.

Usually viral infection is diagnosed if the viral antibody titer is at least four times higher in an acute phase serum sample than that in a sample obtained in the remission phase (with samples collected at least two weeks apart). Although diagnosis of the pathogen in myocarditis is difficult, good diagnostic methods, including a rapid diagnostic test for influenza virus and an RT-PCR assay for influenza H1N1pdm2009 virus from a nasal swab, were already available during the 2009 pandemic. In fact, the prevalence of these new diagnostic methods may be one of the reasons why the number of case reports of myocarditis increased in this pandemic. RT-PCR of the myocardium is more useful for identifying the genomes of viruses causing myocarditis than other methods and showed positive results in 4 patients in our paper [44, 51, 53, 58].

## 7. Treatment of Myocarditis Associated with  Influenza

**Figure 1:**
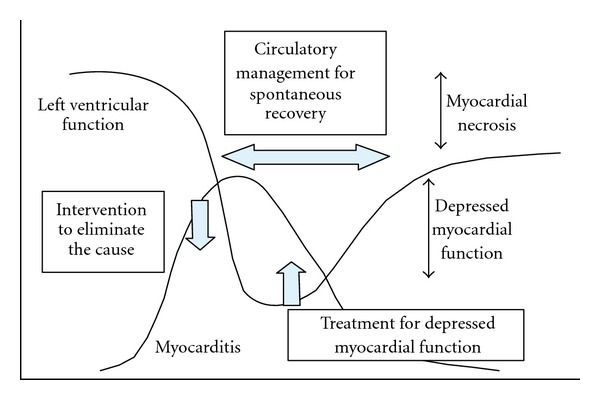
Course of cardiac dysfunction and timing of intervention in myocarditis (Guidelines for Diagnosis and Treatment of Myocarditis (JCS2009)).

The course of cardiac dysfunction and timing of intervention are described in the Guidelines for Diagnosis and Treatment of Myocarditis of the Japan Circulation Society (JCS2009) and are shown in Figure 1. Myocarditis is treated in three ways: (1) intervention to eliminate the cause, (2) intervention to improve hemodynamic compromise, and (3) intervention for cardiac dysfunction [8]. To eliminate the cause, forty-one (85%) of the myocarditis patients in our survey were treated with neuraminidase inhibitors. Treatment with neuraminidase inhibitors is also recommended by the Japanese Association of Infection for all patients infected with influenza [29]. The low-case fatality rate in Japan could be a result of aggressive early intervention with antiviral drugs, such as oseltamivir and zanamivir [28, 29]. Morioka et al. reported no cases of influenza-associated encephalopathy or myocarditis in 44 infants aged <3 months treated with oseltamivir for H1N1pdm2009 infection in Japan [30] and therefore recommended oseltamivir as safe and efficacious for use in infants <3 months of age. Use of immunosuppressive therapy is controversial for both acute myocarditis and influenza infection [8, 24, 27–30, 79, 80]. High-dose steroids are not recommended, because of unproven benefit and potentially harmful effect on influenza infection.

The first therapy for myocarditis patients with heart failure is supportive intervention in potentially fatal cases. The recent application of PCPS and/or IABP in serious cases of viral myocarditis has yielded good outcomes. LVAD or PCPS was used in 10 cases, and IABP was used in 11 cases in this paper. Extracorporeal lung assist (ECMO) was used in 12 cases. These mechanical circulatory devices can be used to decrease mortality and as a bridge to transplantation. Seventeen H1N1pdm2009 myocarditis patients were treated with mechanical circulatory support, thirteen (76%) of whom survived. On the other hand, in the national survey of fulminant myocarditis in Japan, 30 of 52 patients (57.7%) survived without antiviral treatment [10]. Because of the nature of the study design, it was difficult to show that the neuraminidase inhibitors significantly improved the survival rate of patients with fulminant myocarditis associated with influenza. Based on our survey, we recommend that patients with fulminant myocarditis be treated with a combination of neuraminidase inhibitors (to eliminate the causative virus) and mechanical circulatory support (to treat depressed myocardial function).

## 8. Conclusion

We reviewed the details of 58 cases of myocarditis associated with H1N1pdm2009 and found a high prevalence of fulminant myocarditis (36/58, 62%) among them; 14 of these 58 patients died. Diagnosis and treatment during this pandemic were facilitated by improved diagnostic methods (e.g., rapid diagnostic tests, RT-PCR for influenza virus, echocardiogram, and CMR) and by the ready availability of treatment with neuraminidase inhibitors and mechanical circulatory support. We stress the need for increased awareness of influenza-associated myocarditis. Such knowledge will facilitate earlier diagnosis and treatment of this potentially fatal complication during future influenza pandemics.

## Figures and Tables

**Table 1:** Detailed characteristics of 58 patients with myocarditis associated with H1N1pdm2009 influenza.

Characteristics of 58 patients with H1N1pdm2009 influenza reported in detail	Result (%)
Age (mean, years) (range)	32 (3–72)
Less than 17 years (%)	14 cases (24%)
Sex (% female)	30 cases (52%)
Death (%)	14 cases (24%)
Interval between influenza onset and cardiac symptoms (mean, days) (range)	5.4 (1–21)
1st day to the 3rd day (%)	51%
Cardiac symptoms	
Dyspnea (%)	54%
Chest pain (%)	30%
Fulminant myocarditis (%)	36 cases (62%)
Mortality rate of patients with fulminant myocarditis	39% (14/36)
Pneumonia as a complication (%)	13 cases (22%)
ECG findings on the first day of hospitalization	
ST elevation (%)	34%
T inversion (%)	24%
Fatal arrhythmias (VF, VT, complete AV block) (%)	22%
Echocardiogram	
Diffuse or focal left ventricular wall motion abnormalities	90%
Ejection Fraction (mean ± SD)	25 ± 11%
Percentage of patients in whom CAD was ruled out by CAG	41%
Percentage of adult patients in whom CAD was ruled out by CAG	64%
Treatment	
Neuraminidase inhibitors	85%
PCPS	10 cases (17%)
LVAD	1 case (1.7%)
IABP	11 cases (19%)
PCPS or LVAD and/or PCPS	17 cases (29%)
Mortality of patients treated with mechanical support	23% (4/17)
ECMO	12 cases (21%)
Biopsy	10 cases (17%)
Myocarditis with lymphocyte infiltration (mild~moderate)	6 cases
No myocarditis (according to the Dallas criteria)	4 cases
Autopsy	8 cases (14%)
Pachy hemorrhage in the autopsied heart	3/8 cases (38%)
RT-PCR positivity rate for H1N1pdm2009 virus from heart specimens	4 cases

ECG: electrocardiogram; VF: ventricular fibrillation; VT: ventricular tachycardia; AV block: atrioventricular block; CAD: coronary artery disease; CAG: coronary angiography; PCPS: percutaneous cardiopulmonary support; LVAD: left ventricular assist device; IABP: intra-aortic balloon pumping; ECMO: extracorporeal membrane oxygenation; RT-PCR: reverse transcription polymerase chain reaction.
